# Spectrum of practice in the routine management of cervical dystonia with abobotulinumtoxinA: findings from three prospective open-label observational studies

**DOI:** 10.1186/s40734-018-0072-8

**Published:** 2018-07-09

**Authors:** Vijay P. Misra, Richard M. Trosch, Pascal Maisonobe, Savary Om

**Affiliations:** 1 Imperial College Healthcare NHS Trust, London, UK; 2 The Parkinson’s and Movement Disorders Center, 32255 Northwestern Highway, Suite 40, Farmington Hills, MI 48334 USA; 3 Ipsen Pharma, 65 Quai Georges Gorse, 92100 Boulogne-Billancourt, France; 4 Peripheral Nerve Unit, Hammersmith Hospital, London, W12 0HS UK

**Keywords:** Botulinum toxin, Cervical dystonia, Observational study, Meta-analysis

## Abstract

**Background:**

Cervical dystonia is a heterogeneous disorder with several possible presentations, for which first-line therapy is often botulinum toxin (BoNT). In routine clinical practice the success of each BoNT injection is dependent on several variables, including individual presentation and injection technique. Large multicenter, observational studies provide important information on individualized administration strategies that cannot be otherwise ascertained from controlled clinical trials. In this meta-analysis of patient level data, we aimed to evaluate the clinical characteristics of patients with cervical dystonia undergoing routine treatment with botulinum toxin, specifically abobotulinumtoxinA. We also aimed to characterize current abobotulinumtoxinA injection techniques and parameters and to explore international differences in patient presentation and treatment.

**Methods:**

This was a meta-analysis of baseline data from three prospective, international, multicenter, observational studies (NCT01314365, NCT00833196 and NCT01753349) of botulinum toxin treatment for the routine management of adult cervical dystonia.

**Results:**

Data presented illustrate the significant heterogeneity of CD presentation in routine practice. Most subjects presented with a complex pattern of dystonic movements and the majority had additional components of shoulder elevation, tremor and/or jerk. Dosing was generally in accordance with that recommended in the abobotulinumtoxinA prescribing information, although the range of dosing also indicates that injections are tailored to individual presentation. Sub-group analyses at the country level revealed distinct differences in injection practice.

**Conclusions:**

This meta-analysis is based on the largest dataset of subjects with cervical dystonia studied to date. The heterogeneity revealed in our baseline findings support the need to develop consistent, practical and comprehensive best practice guidelines.

## Background

Cervical dystonia (CD) is the most common of the focal dystonias and is characterized by involuntary contractions of the cervical musculature and abnormal movements and postures of the head [1]. It is a heterogeneous disorder, with several possible patterns of head and neck deviations. Dystonic patterns may be ‘simple’, with movements limited to one plane, or ‘complex’, involving more than one plane. Described CD head positions include: torticollis (rotation), laterocollis (tilting), anterocollis (flexion) and retrocollis (extension) [2]. Adding to this clinical complexity, people with CD may also exhibit a variety of additional signs and symptoms, such as shoulder elevation, jerk, neck/shoulder pain and tremor [1–3]. Epidemiological studies have reported a wide range of prevalence estimates (between 28 and 183 cases/million), a female preponderance, and a mean age of onset of 42 years old [4].

Current national and international guidelines recommend chemodenervation with botulinum neurotoxin (BoNT) injections as effective therapy for the management of CD [5, 6]. The American Academy of Neurology currently recommend abobotulinumtoxinA and rimabotulinumtoxinB as having established efficacy and safety (Level A support) and should be offered as a treatment for CD [6]. OnabotulinumtoxinA and incobotulinumtoxinA are supported by Level B evidence and are classified as probably safe and effective for CD [6]. In particular, the recommendations for abobotulinumtoxinA were based on four Class 1 randomized controlled trials (RCTs) [7–10], which have since been supplemented by data from three more trials [11–13]. However, while RCTs remain the preferred study design to inform clinical registration, they can be criticized as they often recruit restricted populations that are not generalizable to the wider patient population. In routine clinical practice, the success of each BoNT injection is dependent on several variables, including individual presentation and injection technique [14, 15]. There is consensus that treatment should be targeted to the primarily affected muscles, and that injectors should consider the optimal concentration (volume of dilution), number of units and number of injections for each muscle to be injected [14]. However, aside from basic dosing recommendations given in the prescribing information, there is only limited information available to guide BoNT injection technique, and as such, decision making is often influenced by external factors such as access to treatment and injector training.

Observational studies, conducted in routine treatment settings, are useful to inform about the current management of CD including the various disease presentations, and allowing for different clinical practices. Such studies also provide important information on individualized administration strategies (e.g. dosing per muscle) that cannot be otherwise ascertained from traditional RCTs. We present here a meta-analysis of patient-level data from three prospective, observational studies to explore how abobotulinumtoxinA is used in the routine treatment of isolated CD. With data from over 1200 subjects (35 countries), it represents the largest dataset for the use of a BoNT-A formulation in CD. As such, it affords unique insights into the range of disease presentations in clinical practice as well as the characterization of current injection techniques, both nationally and internationally.

## Methods

### Description of the studies

The database includes subject baseline data from the three prospective, observational studies which have followed the course of adult CD subjects treated with BoNT-A (Table 1). The methodology and results from the individual studies have been published in detail elsewhere [16–18].

**Table 1:** Studies included in database

Study	Design	Setting	Subjects	Treatment	Key assessments at baseline (1st injection) visit
ANCHOR-CD (NCT01314365) [16]	1-year, non-interventional, registry study	41 sites in the USA	350 adult subjects with CD	AbobotulinumtoxinA (100%)	▪ Demographics ▪ Medical history ▪ AbobotulinumtoxinA injection details ▪ TWSTRS ▪ CDIP-58 ▪ PNRS
INTEREST IN CD1 (NCT00833196) [17]	Non-interventional study following a single BoNT-A injection cycle	38 sites across Australia and Europe	404 adult subjects with CD and a TWSTRS severity score > 15	AbobotulinumtoxinA (69%) OnabotulinumtoxinA (28%) IncobotulinumtoxinA (3%)	▪ Demographics ▪ Medical history ▪ BoNT-A injection details ▪ TWSTRS ▪ Tsui scale (tremor) ▪ CDIP-58
INTEREST IN CD2 (NCT01753349) [18]	3-year, non-interventional study following multiple BoNT-A injection cycles	113 sites across Australia, Europe, Latin America, North Africa, Middle East, Asia, and USA	1050 adult subjects with CD	AbobotulinumtoxinA (69%) OnabotulinumtoxinA (24%) IncobotulinumtoxinA (6%)	▪ Demographics ▪ Medical history ▪ BoNT-A injection details ▪ TWSTRS ▪ Tsui scale (tremor) ▪ Likert scale (patient satisfaction)*

*Only in subjects previously treated with BoNT-A

*BoNT-A* botulinum neurotoxin type A, *CD* cervical dystonia, *CDIP* cervical dystonia impact profile, *PNRS* pain numeric rating scale, *TWSTRS* Toronto Western Spasmodic Torticollis Rating Scale, *USA* United States of America

In each study, the decision to treat was taken prior to, and independently from, the decision to enroll the subject in the study. Subjects could be new to BoNT-A treatment or previously treated with BoNT-A, provided there had been at least a 12-week interval between the last injection and study entry. All subjects in each study underwent a comprehensive clinical CD assessment at baseline/first injection visit. Electronic case report forms were utilized for data collection, including data on medical history, treatment history, and details of the first injection given (e.g. muscles selected, injected dose, injected volume, number of injection sites, use of injection guidance technique). All subjects were also assessed at the baseline/first injection visit using the Toronto Western Spasmodic Torticollis Rating Scale (TWSTRS). The ‘predominant’ patterns of CD were per investigator judgment in the INTEREST IN CD studies and were derived from TWSTRS scores in the ANCHOR-CD study.

### Organization of the database

This meta-analytic database was based on subject level baseline data from three observational studies. No other studies were considered for inclusion into the database as these are the only routine practice studies to prospectively collect injection data with abobotulinumtoxinA. All three studies used electronic case report forms, and data for each subject was checked and monitored at site by the respective Clinical Research Associates. For subjects who participated in two studies (i.e. INTEREST IN CD or ANCHOR-CD and INTEREST IN CD2), only data from the INTEREST IN CD2 study were retained for the meta-analysis. Subject level data from the three studies was merged into a single ‘baseline’ database, and descriptive outcomes were summarized. All outcomes presented in this analysis were assessed and recorded at the first visit (i.e. baseline) of the three studies.

This meta-analysis focuses exclusively on abobotulinumtoxinA because each of the BoNT-A formulations has its own specific recommendations for administration in CD (doses, volume of dilution etc.) and it is well accepted that dose units are specific to the formulation and are not interchangeable [19–21]. As such, dosing and other data for each formulation should be considered separately [22]. Added to this, most subjects across the trials were treated with this formulation (ANCHOR-CD study was restricted to abobotulinumtoxinA) and only 4% of subjects were treated with incobotulinumtoxinA (in only 10 of 35 countries).

### Statistical analyses

Statistical analyses of the pooled baseline data are primarily descriptive. Mean and standard deviation (mean ± SD) or median measures were used to summaries continuous variables, and absolute and relative frequencies expressed as percentage (%) are presented for categorical information. There was no imputation for missing data. Due to concerns that some subjects treated at physiatrist sites may not actually have had isolated CD these baseline analyses excluded 7 physiatrist sites from the original studies (ANCHOR-CD and INTEREST IN CD2). This decision was made because interim analyses and consequent site visits identified that some subjects treated at these sites received bilateral symmetrical injections at equal doses, and injections into several muscles not involved in the dystonic postures.

Recent patient and physician surveys have indicated a range of dosing practices across different countries [23, 24]. We therefore decided to perform exploratory subgroup analyses, using data from countries with reasonable sample sizes (≥4 active sites and data from ≥40 subjects), to look for evidence of heterogeneity of national practice.

## Results

### Subject characteristics

These analyses include baseline data from a total of 1202 subjects with isolated CD treated with abobotulinumtoxinA at 181 neurology centers in 35 countries. Subject demographics, medical history, and clinical severity scores at Visit 1 (baseline) are presented in Table 2. Most subjects (66%) were female and 86% were aged at least 41 years old.

**Table 2:** Demographic, medical history and clinical characteristics at baseline

Characteristic	AbobotulinumtoxinA treated subjects (*N* = 1202)
Sex (female); n (%)	797 (66.3)
Age; n (%)	
18–30	50 (4.2)
31–40	118 (9.8)
41–50	261 (21.7)
51–60	306 (25.5)
61–70	295 (24.5)
> 70	172 (14.3)
Proportion subjects with CD family history; n (%)	85 (7.1)
Time since diagnosis (years); n (%)	
< 1	149 (12.4)
1–5	415 (34.6)
> 5	637 (53.0)
Missing	1
Previous treatment with BoNT; n (%)	
Yes	994 (82.8)
No	206 (17.2)
Missing	2
Use of concomitant medication; n (%)	453 (37.7)
Proportion with simple CD; n (%)*	373 (31.1)
Proportion with complex CD; n (%)	821 (68.4)
Predominant head/neck deviation pattern; n (%)	
Torticollis	758 (64.1)
Laterocollis	243 (20.5)
Retrocollis	57 (4.8)
Anterocollis	24 (2.0)
Lateral shift	11 (0.9)
Sagittal shift	10 (0.8)
Missing/not derived	99
Secondary head/neck deviation pattern and associated components; n (%)	
Torticollis	283 (34.3)
Laterocollis	468 (56.7)
Retrocollis	200 (24.2)
Anterocollis	151 (18.3)
Lateral shift	170 (20.6)
Sagittal shift	78 (9.5)
No secondary pattern	68 (5.7)
Missing/not derived	309
CD component; n (%)	
Shoulder elevation; n (%)	499 (71.6)
Tremor; n (%)	393 (56.4)
Jerk; n (%)	86 (12.3)
No component	164
Missing	341
Tsui tremor score category; n (%)	
0	445 (47.2)
1	238 (25.3)
2	155 (16.5)
4	104 (11.0)
Missing	260
Tsui tremor severity category; n (%)	
None	445 (47.2)
Mild	381 (40.4)
Severe	116 (12.3)
Missing	260
Tsui tremor duration category; n (%)	
None	445 (47.2)
Occasional	250 (26.5)
Continuous	247 (26.2)
Missing	260
TWSTRS Total; mean ± SD**	33.95 ± 12.46
TWSTRS Severity; mean ± SD**	17.18 ± 5.13
TWSTRS Disability; mean ± SD**	9.95 ± 6.30
TWSTRS Pain; mean ± SD**	6.82 ± 4.94

*6 subjects had no pattern. **Missing data for one subject

*BoNT* botulinum neurotoxin, *CD* cervical dystonia, *SD* standard deviation, *TWSTRS* Toronto Western Spasmodic Torticollis Rating Scale

The five countries with largest sample sizes were: France (*n* = 118), Germany (*n* = 95), Russia (*n* = 96), United Kingdom (UK; *n* = 59) and United States of America (USA; *n* = 277). Roughly seven in ten subjects (68.4%) had a complex presentation of CD (defined as having more than one pattern of CD). The most common predominant head/neck patterns were torticollis and laterocollis.

### Injection parameters

Data for the abobotulinumtoxinA injection parameters are summarized in Table 3. The median dose of abobotulinumtoxinA was consistently 500 U, the full range of doses indicated that injectors tailor doses to individual patient presentations. However, subgroup analyses at the country level revealed several national differences in injection practice. For example, while the maximum total dose given in Russia was 1000 U, some subjects in Germany and the USA received higher total doses of 1500–1600 U. Likewise, the median number of injection placement sites in the UK and France were observably lower than in Russia, the USA and Germany despite the median number of muscles injected being similar.

**Table 3:** Administration of abobotulinumtoxinA at baseline (Visit 1) by country and overall

Parameter	Overall (N = 1202)*	France (N = 118)	Germany (N = 95)	Russia (N = 96)	UK (N = 59)	USA (N = 277)
Total aboBoNT-A dose (U)						
Mean ± SD	563 ± 245	571 ± 159	647 ± 270	674 ± 219	503 ± 163	564 ± 235
Median [range]	500 [50–1700]	500 [200–1000]	500 [120–1600]	500 [250–1000]	500 [150–1150]	500 [100–1502]
Number of muscles injected; median [range]	3 [1–11]	3 [1–6]	4 [1–10]	4 [2–9]	3 [1–6]	5 [1–18]
Most commonly injected muscles; n (% subjects)						
Splenius capitis	1087 (90.4)	113 (95.8)	87 (91.6)	95 (99.0)	52 (88.1)	249 (89.9)
Sternocleidomastoid	967 (80.4)	102 (86.4)	83 (87.4)	95 (99.0)	44 (74.6)	196 (70.8)
Trapezius	739 (61.5)	54 (45.8)	44 (46.3)	82 (85.4)	32 (54.2)	155 (56.0)
Levator scapulae	569 (47.3)	39 (33.1)	55 (57.9)	52 (54.2)	28 (47.5)	174 (62.8)
Semispinalis capitis	334 (27.8)	24 (20.3)	36 (37.9)	23 (24.0)	11 (18.6)	126 (45.5)
Scalene group**	164 (13.8)	4 (3.4)	14 (14.7)	9 (9.4)	0	53 (19.1)
Longissimus group**	86 (7.2)	4 (3.4)	4 (4.2)	0	0	57 (20.6)
Splenius cervicis	60 (5.0)	1 (0.8)	3 (3.2)	0	0	44 (15.9)
Obliquus capitis inferior	42 (3.5)	1 (0.8)	7 (7.4)	1 (1.0)	0	19 (6.9)
Platysma	24 (2.0)	1 (0.8)	5 (5.3)	5 (5.2)	2 (3.4)	0
Number of injection points; median [range]	7.0 [1–34]	4.5 [1–16]	8.0 [2–27]	9.5 [4–28]	4.0 [1–6]	9.0 [1–34]
Injected volume (mL); median [range]	2.00 [0.2–12.0]	1.25 [0.6–5.0]	2.50 [0.6–8.0]	2.00 [0.5–5.0]	2.25 [0.7–4.6]	2.00 [0.2–12.0]
Use of injection guidance; n (%)	486 (40.5)	50 (42.4)	68 (71.6)	12 (12.5)	1 (1.7)	166 (59.9)
Interval since last injection (days);***	n/*N* = 385/849	n/*N* = 47/91	n/*N* = 20/62	n/*N* = 31/76	n/*N* = 19/54	n/*N* = 78/114
Median [range]	108.3 [15.0–317.7]	108.9 [68.8–284.2]	99.5 [57.7–214.0]	112.5 [76.9–256.5]	104.2 [50.8–198.0]	94.3 [15.0–225.0]

* Missing data for one subject. **Scalene group includes muscles reported in the eCRF as scalenus or scalene (medius, anterior and/or posterior). Longissimus group includes muscles reported in the eCRF as longissimus, longissimus capitis and/or longissimus cervicis. ***Injection prior to study entry, where N = subjects previously treated with abobotulinumtoxinA only

*AboBoNT-A* abobotulinumtoxinA, *eCRF* electronic case report form, *mL* milliliter, *SD* standard deviation, *U* units, *UK* United Kingdom, *USA* United States of America

Overall, less than half (41%) of subjects were injected for CD using a guidance technique; the most commonly used technique was electromyography (EMG; used in 39% of subjects) followed by ultrasonography (3%) and electrostimulation (1%). Overall, EMG was used in 467 of the 486 injections (96%) given using a guidance technique. Of note, injectors in Germany and the USA were at least five times more likely to use an injection guidance technique than those in Russia and the UK. Analysis of time to retreatment between last injection prior to study entry and baseline injection visit for those subjects previously treated with abobotulinumtoxinA (data available for 385 out of 849 subjects previously treated with abobotulinumtoxinA), found that the median interval for re-treatment was 15.5 weeks, although some subjects had much longer intervals of up to 45 weeks.

The most commonly injected muscles were the splenius capitis (injected in 91% of subjects), sternocleidomastoid (81%), trapezius (62%), levator scapulae (47%), semispinalis capitis (28%) and scalene group (14%); all other relevant muscles were injected in < 10% of subjects. These were the most commonly injected muscles regardless of whether the subject had a complex or simple presentation. Subgroup analyses at the country level identified some national trends. For example, subjects in Russia were more likely to receive injections into the trapezius (85%) than subjects in France and Germany (46%). In Germany and the USA, subjects were twice as likely to receive injections into the semispinalis capitis (38 and 46%, respectively) than those in the other countries (19–24%). Whereas injectors in the US performed scalene and longissimus group injections in about 20% of subjects, injectors in the UK did not inject into these muscles at all. The injection parameters in the top 10 injected muscles with abobotulinumtoxinA are provided in Table 4.

**Table 4:** Administration of abobotulinumtoxinA at baseline by muscle

Muscle	AbobotulinumtoxinA treated subjects (*N* = 1202)
Splenius capitis	*N* = 1087
Dose (U); mean ± SD	226 ± 134
Dose (U); median [range]	200 [10–1100]
Volume (mL); median [range]	0.75 [0.1–4.4]
Number of injection points; median [range]	2 [1–12]
Use of injection guidance (%)*	40.1
Sternocleidomastoid	*N* = 967
Dose (U); mean ± SD	147 ± 81
Dose (U); median [range]	130 [15–600]
Volume (mL); median [range]	0.50 [0.1–2.8]
Number of injection points; median [range]	2 [1–10]
Use of injection guidance (%)*	38.0
Trapezius	*N* = 739
Dose (U); mean ± SD	163 ± 111
Dose (U); median [range]	140 [10–1000]
Volume (mL); median [range]	0.50 [0.1–5.3]
Number of injection points; median [range]	2 [1–12]
Use of injection guidance (%)*	34.9
Levator scapulae	*N* = 569
Dose (U); mean ± SD	125 ± 82
Dose (U); median [range]	100 [13–550]
Volume (mL); median [range]	0.40 [0.1–2.8]
Number of injection points; median [range]	1 [1–10]
Use of injection guidance (%)*	48.9
Semispinalis capitis	*N* = 334
Dose (U); mean ± SD	138 ± 84
Dose (U); median [range]	100 [10–500]
Volume (mL); median [range]	0.50 [0.1–2.2]
Number of injection points; median [range]	2 [1–8]
Use of injection guidance (%)*	49.4
Scalene group**	*N* = 164
Dose (U); mean ± SD	100 ± 68
Dose (U); median [range]	85 [13–450]
Volume (mL); median [range]	0.30 [0.1–1.5]
Number of injection points; median [range]	2 [1–8]
Use of injection guidance (%)*	50.0
Longissimus group***	*N* = 86
Dose (U); mean ± SD	128 ± 66
Dose (U); median [range]	100 [20–300]
Volume (mL); median [range]	0.40 [0.0–1.2]
Number of injection points; median [range]	2 [1–8]
Use of injection guidance (%)*	64.0
Splenius cervicis	*N* = 60
Dose (U); mean ± SD	122 ± 79
Dose (U); median [range]	100 [20–500]
Volume (mL); median [range]	0.50 [0.1–1.6]
Number of injection points; median [range]	1 [1–11]
Use of injection guidance (%)*	65.0
Obliquus capitis inferior	*N* = 42
Dose (U); mean ± SD	75 ± 44
Dose (U); median [range]	60 [20–250]
Volume (mL); median [range]	0.30 [0.1–1.3]
Number of injection points; median [range]	1 [1–2]
Use of injection guidance (%)*	93.0
Platysma	*N* = 24
Dose (U); mean ± SD	72 ± 33
Dose (U); median [range]	60 [30–170]
Volume (mL); median [range]	0.25 [0.1–0.9]
Number of injection points; median [range]	4 [1–12]
Use of injection guidance (%)*	12.5

*Denominator is the number of injections into the muscle. **Scalene group includes muscles reported in the eCRF as scalenus or scalene (medius, anterior and/or posterior). ***Longissimus group includes muscles reported in the eCRF as longissimus, longissimus capitis and/or longissimus cervicis

*eCRF* electronic case report form, *mL* milliliter, *SD* standard deviation, *U* units

## Discussion

This database represents the largest cohort of CD patients ever followed and provides a unique insight into how abobotulinumtoxinA is used in routine clinical practice for a broad spectrum of patients being treated for CD.

We have used baseline data to describe how patients present when they attend a routine clinic visit for ongoing or de novo treatment with abobotulinumtoxinA, and the findings showcase the significant heterogeneity of how BoNT-A is used in routine practice. Of note, the relatively unrestrictive entry criteria to the observational studies allowed us to include subjects with a broader spectrum of disease than most RCTs. This is highlighted by lower mean TWSTRS Total scores (33.95 in the present analysis vs. 43.23 in RCTs) [25], supporting the idea that RCTs often recruit subjects with more severe disease [25]. Moreover, we also observed that most patients presented with a complex pattern of dystonic movements and the majority had additional components of shoulder elevation, tremor and/or jerk. This is also in contrast to some clinical studies which have preferentially recruited patients with torticollis [26, 27], and suggests that such trial designs might have limited generalizability. As such, we suggest that large observational studies alongside RCTs have a significant place in evaluating the clinical effectiveness of a treatment in routine practice.

Dosing was generally in accordance with that recommended in the abobotulinumtoxinA prescribing information [19, 28]. The median dose was 500 U, which is also the recommended starting dose for abobotulinumtoxinA, and evidence from long-term open-label studies have suggested that most patients with isolated CD continue to benefit from this dosing regimen over repeated cycles [29]. Nevertheless, clinical guidelines recommend that doses must be tailored to the patient’s individual presentation, and injection parameters should be based on considerations of types of muscle (i.e. which movements they mediate), degree of abnormal muscle activity and muscle size [14]. While INTEREST in CD1 was a single cycle study, both INTEREST in CD2 and ANCHOR-CD allowed injectors to change the dosing parameters and/or muscles injected according to patient response. Future analyses of the combined database will evaluate the evolution of dosing over 1 year of repeat treatments. Nevertheless, these baseline data strongly suggests that many injectors are comfortable tailoring treatment, with some injectors going outside of the recommended dosing range of 250–1000 U. Here, the use of total doses above 1000 U seems to be influenced by national factors with, for example, Russian injectors never going outside of the recommended dose range. Average doses per muscle were also in general alignment with current prescribing information, which provides basic guidance for injection [19, 28]. In particular, it has been suggested that unilateral injections of abobotulinumtoxinA with doses above 150 U into the sternocleidomastoid muscle is associated with a higher dysphagia risk [29], and the median dose injected into this muscle was 130 U. However, it should be noted that the range of sternocleidomastoid dosing extended to 600 U. In this respect, it is important to note that these analyses describe how abobotulinumtoxinA is currently utilized in CD, but not how abobotulinumtoxinA should be ideally utilized in CD. Studies comparing the effectiveness of different muscle selection and injection parameters would be needed to address this issue.

Across all countries, the most commonly injected muscles were also those most easily accessible. While these muscles are largely appropriate for torticollis and laterocollis, it is unknown if these common selections are the best choices in every patient. At the country level, our findings also revealed some differences in the frequency that certain muscles were chosen. For example, subjects in Germany and the USA appeared more likely to receive injections into the semispinalis capitis, levator scapulae and other smaller, deeper cervical muscles than other countries. This highlights the need to investigate whether injection of these muscles improves outcome. If so, more work through available training programs [30] will be required to standardize treatment paradigms. Of note, injectors in these two countries were also more likely to use injection guidance than other countries and one could speculate whether the availability (or training levels) of injection guidance techniques influences choice of muscle selection.

The most commonly injected muscles are superficial, large and easy to palpate and this may be the reason that use of injection guidance was less than 40% overall. While many injectors believe that injection guidance is not necessary for simple torticollis, it has been suggested that use of EMG to identify target muscles and guide injections is important for patients with complex forms of CD, and especially in so called ‘non-responders’ or those who have suffered adverse events (e.g. related to toxin spread to adjacent muscles) [31, 32]. Using EMG guidance allows the injector to isolate the muscle fibers that are actively contracting (vs. those which are quiescent) and contributing to the dystonia. One open label trial reported that use of EMG improved the treatment outcome in 9 out of 20 subjects who had been initially identified as secondary non-responders [33]. Another study in five subjects found that use of ultrasound to guide BoNT-A injections into the sternocleidomastoid muscle eliminated the dysphagia which had previously limited the use of BoNT-A in these subjects [34]. Injection guidance is also suggested as necessary to improve accuracy of injections into the deeper or thinner neck muscles [35–37]. This advice appears to be well followed for some of the most difficult to target muscles such as the obliquus capitis inferior, for which 93% of the 42 injections recorded were given under guidance (EMG and/or ultrasonography). However, it was not as commonly used for injections into other relatively thin muscles such as the sternocleidomastoid (< 40% of injections). In their small study, Hong et al. found that the average thickness of the sternocleidomastoid is less than 1.1 cm (patients and controls), and suggested that potential contributors to dysphagia could be an underestimation of needle depth leading to either injection into non-target muscles or remote spread through the fascial borders of the sternocleidomastoid into the deeper neck muscles [34].

Strengths of our analyses are based on its size and international reach. However, since the database includes data from subjects treated in 35 different countries, it is highly likely that the range of data reflects international differences in several factors, including access to treatment, service reimbursement (e.g. private or state funded), specialist training for injectors and patient preference. This analysis was limited to abobotulinumtoxinA, but other observational studies have confirmed the clinical utility of the other available formulations [38, 39]. We made the decision to exclude physiatrist sites, as site visits identified that some subjects at these centers were being treated for non-dystonic conditions causing neck pain. These few subjects were included in the primary analyses of the observational studies where the objective was to report the routine use of BoNT, but excluded here because we wanted to provide clear and detailed information on the dosing of abobotulinumtoxinA for isolated CD. Our analyses at this baseline stage were descriptive and no statistical comparisons were pre-planned. Future work can build on this exploratory work and test for hypothesized country-level differences in injection practices. Other limitations include those inherent to observational studies (e.g. level of missing data), as well as the recruitment of smaller subject numbers in many countries and potential site selection bias.

## Conclusions

This meta-analysis of baseline data is based on the largest dataset of subjects with CD studied to date. We have only presented a ‘snapshot’ of clinical practice, but the database will continue to collect longitudinal data over several treatment cycles (repeat cycle data from INTEREST IN CD2 and ANCHOR-CD) allowing exploration of how treatment variables affect patient outcomes. For example, longitudinal analyses will allow exploration of whether different injection parameters (e.g. lower vs. higher doses, single vs. multiple injection points per muscle, small vs. large dilution volumes, use of injection guidance etc.) provide any observable benefit in terms of impact upon disease severity or treatment satisfaction. Overall, the range of our baseline findings support the need to develop consistent, practical and comprehensive best practice guidelines.

## Abbreviations

AboBoNT-A: abobotulinumtoxinA
BoNT-A: Botulinum neurotoxin type A
CD: Cervical dystonia
CDIP: Cervical dystonia impact profile
eCRF: Electronic case report form
EMG: Electromyography
PNRS: Pain numeric rating scale
RCT: Randomized controlled trial
TWSTRS: Toronto Western Spasmodic Torticollis Rating Scale
U: Units
UK: United Kingdom
USA: United States of America

## References

[1] Albanese A, Bhatia K, Bressman SB, Delong MR, Fahn S, Fung VS (2013). Phenomenology and classification of dystonia: a consensus update. Mov Disord.

[2] Reichel G (2011). Cervical dystonia: a new phenomenological classification for botulinum toxin therapy. Basal Ganglia.

[3] Jankovic J, Tsui J, Bergeron C (2007). Prevalence of cervical dystonia and spasmodic torticollis in the United States general population. Parkinsonism Relat Disord.

[4] Defazio G, Jankovic J, Giel JL, Papapetropoulos S. Descriptive epidemiology of cervical dystonia. Tremor Other Hyperkinet Mov (N Y). 2013;3 tre-03-193-4374-2 10.7916/D80C4TGJ PMC3822401 24255801

[5] Albanese A, Asmus F, Bhatia KP, Elia AE, Elibol B, Filippini G (2011). EFNS guidelines on diagnosis and treatment of primary dystonias. Eur J Neurol.

[6] Simpson DM, Hallett M, Ashman EJ (2016). Practice guideline update summary: botulinum neurotoxin for the treatment of blepharospasm, cervical dystonia, adult spasticity, and headache. Neurology.

[7] Poewe W, Deuschl G, Nebe A, Feifel E, Wissel J, Benecke R, German Dystonia Study Group (1998). What is the optimal dose of botulinum toxin a in the treatment of cervical dystonia? Results of a double blind, placebo controlled, dose ranging study using Dysport. J Neurol Neurosurg Psychiatry.

[8] Wissel J, Kanovsky P, Ruzicka E, Bares M, Hortova H, Streitova H (2001). Efficacy and safety of a standardised 500 unit dose of Dysport (clostridium botulinum toxin type a haemaglutinin complex) in a heterogeneous cervical dystonia population: results of a prospective, multicentre, randomised, double-blind, placebo-controlled, parallel group study. J Neurol.

[9] Truong D, Brodsky M, Lew M, Brashear A, Jankovic J, Molho E (2010). Long-term efficacy and safety of botulinum toxin type a (Dysport) in cervical dystonia. Parkinsonism Relat Disord.

[10] Truong D, Duane DD, Jankovic J, Singer C, Seeberger LC, Comella CL (2005). Efficacy and safety of botulinum type a toxin (Dysport) in cervical dystonia: results of the first US randomized, double-blind, placebo-controlled study. Mov Disord.

[11] Poewe W, Burbaud P, Castelnovo G, Jost WH, Ceballos-Baumann AO, Banach M (2016). Efficacy and safety of abobotulinumtoxinA liquid formulation in cervical dystonia: a randomized-controlled trial. Mov Disord.

[12] Lew M, Snyder D. E Efficacy and safety of a 2 mL dilution of abobotulinumtoxinA compared with placebo in adult patients with cervical dystonia. Mov Disord. 2017;32(suppl 2) http://www.mdsabstracts.org/abstract/efficacy-and-safety-of-a-2-ml-dilution-of-abobotulinumtoxina-compared-with-placebo-in-adult-patients-with-cervical-dystonia/. Accessed 8 Nov 2017 10.1016/j.pmrj.2016.07.463 27673045

[13] Yun JY, Kim JW, Kim HT, Chung SJ, Kim JM, Cho JW (2015). Dysport and Botox at a ratio of 2.5:1 units in cervical dystonia: a double-blind, randomized study. Mov Disord.

[14] Albanese A, Abbruzzese G, Dressler D, Duzynski W, Khatkova S, Marti MJ, et al. Practical guidance for CD management involving treatment of botulinum toxin: a consensus statement. J Neurol. 2015;262(10):2201–13. 10.1007/s00415-015-7703-x PMC4608989 25877834

[15] Marion MH, Humberstone M, Grunewald R, Wimalaratna S (2016). British neurotoxin network recommendations for managing cervical dystonia in patients with a poor response to botulinum toxin. Pract Neurol.

[16] Trosch R, Espay A, Truong D, Gil R, Singer C, LeWitt PA (2017). Multicenter observational study of abobotulinumtoxinA neurotoxin in cervical dystonia: the ANCHOR-CD registry. J Neurol Sci.

[17] Misra VP, Ehler E, Zakine B, Maisonobe P, Simonetta-Moreau M. Factors influencing response to botulinum toxin type a in patients with idiopathic cervical dystonia: results from an international observational study. BMJ Open. 2012;2(3):e000881. 10.1136/bmjopen-2012-000881 PMC3378940 22700836

[18] Misra VP, Colosimo C, Charles D, Chung TM, Maisonobe P, Om S (2018). INTEREST IN CD2, a global patient-centred study of long-term cervical dystonia treatment with botulinum toxin. J Neurol.

[19] Dysport Summary of Product Characteristics. www.medicines.org.uk/emc/product/859/smpc. (last accessed June 2018).

[20] Xeomin - Summary of Product Characteristics. www.medicines.org.uk/emc/product/2162/smpc. (last accessed June 2018).

[21] Botox 100 Units - Summary of Product Characteristics www.medicines.org.uk/emc/product/859/smpc. (last accessed June 2018).

[22] Pickett A, Rosales RL (2011). New trends in the science of botulinum toxin-a as applied in dystonia. Int J Neurosci.

[23] Comella C, Bhatia K (2015). An international survey of patients with cervical dystonia. J Neurol.

[24] Hubble J, Schwab J, Hubert C, Abbott CC (2013). Dysport (botulinum toxin type A) in routine therapeutic usage: a telephone needs assessment survey of European physicians to evaluate current awareness and adherence to product labeling changes. Clin Neuropharmacol.

[25] Jen MH, Kurth H, Iheanacho I, Dinet J, Gabriel S, Wasiak R (2014). Assessing the burden of illness from cervical dystonia using the Toronto western spasmodic torticollis rating scale scores and health utility: a meta-analysis of baseline patient-level clinical trial data. J Med Econ.

[26] Comella CL, Jankovic J, Truong DD, Hanschmann A, Grafe S, Group USXCDS (2011). Efficacy and safety of incobotulinumtoxinA (NT 201, XEOMIN(R), botulinum neurotoxin type a, without accessory proteins) in patients with cervical dystonia. J Neurol Sci.

[27] Benecke R, Jost WH, Kanovsky P, Ruzicka E, Comes G, Grafe S (2005). A new botulinum toxin type a free of complexing proteins for treatment of cervical dystonia. Neurology.

[28] Dysport (abobotulinumtoxinA) for injection, for intramuscular use. Full US prescribing information. Available at http://dysport.com. Last accessed June 2018.

[29] Hauser RA, Truong D, Hubble J, Coleman C, Beffy JL, Chang S (2013). AbobotulinumtoxinA (Dysport) dosing in cervical dystonia: an exploratory analysis of two large open-label extension studies. J Neural Transm.

[30] Fheodoroff K, Bhidayasiri R, Jacinto LJ, Chung TM, Bhatia K, Landreau T (2017). Ixcellence network(R): an international educational network to improve current practice in the management of cervical dystonia or spastic paresis by botulinum toxin injection. Funct Neurol.

[31] Nijmeijer SW, Koelman JH, Kamphuis DJ, Tijssen MA (2012). Muscle selection for treatment of cervical dystonia with botulinum toxin--a systematic review. Parkinsonism Relat Disord.

[32] Van Gerpen JA, Matsumoto JY, Ahlskog JE, Maraganore DM, McManis PG (2000). Utility of an EMG mapping study in treating cervical dystonia. Muscle Nerve.

[33] Cordivari C, Misra VP, Vincent A, Catania S, Bhatia KP, Lees AJ (2006). Secondary nonresponsiveness to botulinum toxin a in cervical dystonia: the role of electromyogram-guided injections, botulinum toxin a antibody assay, and the extensor digitorum brevis test. Mov Disord.

[34] Hong JS, Sathe GG, Niyonkuru C, Munin MC (2012). Elimination of dysphagia using ultrasound guidance for botulinum toxin injections in cervical dystonia. Muscle Nerve.

[35] Truong DMD, Dressler D, Hallett M, Pathak M (2009). Manual of botulinum toxin therapy.

[36] Lee IH, Yoon YC, Sung DH, Kwon JW, Jung JY (2009). Initial experience with imaging-guided intramuscular botulinum toxin injection in patients with idiopathic cervical dystonia. AJR Am J Roentgenol.

[37] Schramm A, Baumer T, Fietzek U, Heitmann S, Walter U, Jost WH (2015). Relevance of sonography for botulinum toxin treatment of cervical dystonia: an expert statement. J Neural Transm (Vienna).

[38] Jankovic J, Adler CH, Charles D, Comella C, Stacy M, Schwartz M (2015). Primary results from the cervical dystonia patient registry for observation of onabotulinumtoxina efficacy (CD PROBE). J Neurol Sci.

[39] Fernandez HH, Pagan F, Danisi F, Greeley D, Jankovic J, Verma A, et al. Prospective study evaluating incobotulinumtoxinA for cervical dystonia or blepharospasm: Interim results from the first 145 subjects with cervical dystonia. Tremor Other Hyperkinet Mov. 2013;3 tre-03-139-2924-1 10.7916/D8CF9NVD PMC3638085 23724362

